# Leptin/HER2 crosstalk in breast cancer: *in vitro* study and preliminary *in vivo* analysis

**DOI:** 10.1186/1471-2407-8-305

**Published:** 2008-10-22

**Authors:** Elena Fiorio, Anna Mercanti, Marianna Terrasi, Rocco Micciolo, Andrea Remo, Alessandra Auriemma, Annamaria Molino, Veronica Parolin, Bruno Di Stefano, Franco Bonetti, Antonio Giordano, Gian Luigi Cetto, Eva Surmacz

**Affiliations:** 1 Sbarro Institute for Cancer Research and Molecular Medicine, Temple University, Philadelphia, USA; 2 Department of Medical Oncology, University of Verona, Italy; 3 Department of Anatomic Pathology, University of Verona, Italy; 4 Section of Medical Oncology, Department of Surgical Oncology, University of Palermo, Italy; 5 Department of Sociology and Social Research, University of Trento, Italy

## Abstract

**Background:**

Obesity in postmenopausal women is associated with increased breast cancer risk, development of more aggressive tumors and resistance to certain anti-breast cancer treatments. Some of these effects might be mediated by obesity hormone leptin, acting independently or modulating other signaling pathways. Here we focused on the link between leptin and HER2. We tested if HER2 and the leptin receptor (ObR) can be coexpressed in breast cancer cell models, whether these two receptors can physically interact, and whether leptin can transactivate HER2. Next, we studied if leptin/ObR can coexist with HER2 in breast cancer tissues, and if presence of these two systems correlates with specific clinicopathological features.

**Methods:**

Expression of ObR, HER2, phospo-HER2 was assessed by immonoblotting. Physical interactions between ObR and HER2 were probed by immunoprecipitation and fluorescent immunostaining. Expression of leptin and ObR in breast cancer tissues was detected by immunohistochemistry (IHC). Associations among markers studied by IHC were evaluated using Fisher's exact test for count data.

**Results:**

HER2 and ObR were coexpressed in all studied breast cancer cell lines. In MCF-7 cells, HER2 physically interacted with ObR and leptin treatment increased HER2 phosphorylation on Tyr 1248. In 59 breast cancers, the presence of leptin was correlated with ObR (the overall association was about 93%). This result was confirmed both in HER2-positive and in HER2-negative subgroups. The expression of leptin or ObR was numerically more frequent in larger (> 10 mm) tumors.

**Conclusion:**

Coexpression of HER2 and the leptin/ObR system might contribute to enhanced HER2 activity and reduced sensitivity to anti-HER2 treatments.

## Background

Recent epidemiological and clinical data confirmed that obesity in postmenopausal women is associated with increased breast cancer risk, development of more aggressive breast tumors and resistance to certain anti-breast cancer treatments [1-4]. The molecular mechanisms of this link are not clear, but several studies in animal and cellular models suggested that excess body weight could promote breast cancer through increased production of an adipocyte-derived hormone leptin [5-7]. The primary function of leptin is to regulate energy balance and food intake by acting in the brain, but the hormone also plays an important role in peripheral organs, modulating fertility, lactation, and immune response [8,9]. Leptin levels in humans correlate with adiposity and are usually higher in females than in males [8].

Leptin action is mediated through the transmembrane leptin receptor ObR [10]. The human ObR can be expressed as at least four isoforms with different COOH-terminal cytoplasmic domains [11]. The full (long) form of ObR (ObRl) contains the extracellular, transmembrane, and intracellular domains [10]. Only ObRl has a full signaling potential, while the short ObR isoforms (ObRs) have diminished or abolished signaling activity [12]. ObRl is highly expressed in the hypothalamus, however lower levels of ObRl have been identified in many peripheral tissues [5,13-15]. The major pathways activated by ObRl are the classic cytokine JAK2/STAT3 pathway, the Ras/ERK1/2 signaling cascade, and the PI-3K/Akt/GSK3 growth/anti-apoptotic pathway [12].

Recently, leptin has been found to be involved in neoplastic processes, especially in breast carcinogenesis [5-7,16]. Specifically, leptin can promote cancer cell growth and transformation in vitro and in vivo, and increase cell survival in the presence of anti-cancer drugs [5,17]. The role of leptin in breast cancer has been substantiated by the fact breast tumors, but not normal mammary epithelium, overexpress both leptin and ObR [18-20], and the leptin/ObR system correlates with higher tumor grade and worse prognosis [18,19]. In addition, intratumoral levels of ObRl and ObRs and high levels of serum leptin were found to correlate with poor prognosis [21].

Leptin may exert its activity not only through ObR, but also through crosstalks with other signaling systems. For instance, leptin affects the synthesis and/or function of estrogen receptor alpha (ERα), vascular endothelial growth factor (VEGF), and human epidermal growth factor receptor 2 (HER2) [5,6,22-25]. Leptin may also promote tumor cell survival in xenograft models via increased expression of E-cadherin [17].

HER2 is a tyrosine kinase that is amplified in 25–30% of breast tumors and its overexpression often correlates with a more aggressive, metastatic phenotype and worse prognosis [26,27]. Current therapies of HER2-positive tumors include treatments with trastuzumab (Herceptin), a monoclonal HER2 antibody, but resistance to this drug is a common problem that ultimately leads to treatment failure [28]. The molecular basis of trastuzumab resistance are still obscure, but there is evidence that increased activation of other growth factor signaling systems may contribute to this process [28].

Preliminary data obtained in human embryonic kidney HEK 293T cells engineered to coexpress HER2 and ObRs or ObRl suggested that leptin, acting through either ObR isoform, can rapidly induce HER2 phosphorylation and activation of HER2 intracellular signaling [22]. Recent data of Soma et al. suggested that in SK-BR-3 breast cancer cells, leptin can transactivate HER2 through both the epidermal growth factor receptor HER1 and JAK2 pathways [29]. Thus, transactivation of HER2 by liganded ObR or by HER1 might constitute an important mechanism of HER2 resistance in breast cancer patients, especially those expressing high levels of leptin and ObR in breast cancer tissues. However, the existence of functional leptin/HER2 interactions in human breast cancer has not been explored.

Consequently, using breast cancer cell lines naturally expressing HER2 and ObR, we tested if HER2 and ObR can physically interact and if exposure of cells to leptin can transactivate HER2. To validate in vitro data, we studied whether the leptin/ObR system and HER2 can be coexpressed in breast cancer biopsies and if coexpression of these two systems is associated with specific clinicopathological features.

## Methods

### Patients and tissue samples

The expression of leptin, ObR, and other markers was assessed in breast cancer samples. Tissue samples were obtained from 59 women (31 HER2-positive and 28 HER2-negative) who underwent partial or total mastectomy and lymph node dissection for primary breast cancer at the University and Public Hospitals in Verona between January 1, 1992 and November 15, 2006 (Table. 1). All of the patients had a histologically confirmed diagnosis of breast cancer. Patients with a histological diagnosis of breast sarcomas and males with breast cancer were excluded from the analysis. Clinical staging was applied according to the sixth edition of the Union International Contre le Cancer/American Joint Committee on Cancer TNM classification manual [30]. All tissue samples were anonymized and the local ethical committee approved the study protocol.

**Table 1:** Patient and tumor characteristics

**Clinicopathological Features**	**Patients n (%)**	**Clinicopathological Features**	**Patients n (%)**
***Menopausal status***		T	
Postmenopausal	15 (25)	TIS	3 (5)
Premenopausal	39 (66)	pTx	1 (2)
Unknown	5 (9)	pT1	19 (33)
***Histotype***		pT2	22 (37)
Ductal invasive	45 (76)	pT3	2 (3)
Lobular invasive	4 (7)	pT4	12 (20)
Intraductal	3 (5)	***Diameter (mm)***	
Inflammatory	4 (7)	≤ 10 mm	9 (15)
Other	3 (5)	> 10 mm	42 (71)
***Grading***		Unknown	8 (14)
G1	7 (12)		
G2	18 (31)	***N***	
G3	26 (44)	pN1-3	20 (34)
Unknown	8 (13)	pN4-10	9 (15)
Ki-67		pN > 10	4 (7)
0–15%	33 (56)	Negative	19 (32)
16–25%	9 (15)	Unknown	7 (12)
26–100%	14 (24)	HER-2	
Unknown	3 (5)	Positive	31 (53)
***ER/PgR***		Negative	28 (47)
ER+/PgR+	38 (64)		
ER-/PgR-	12 (20)	***LVI***	
ER+/PgR-	7 (12)	Positive	27 (46)
ER-/PgR+	1 (2)	Negative	24 (41)
Unknown	1 (2)	Unknown	8 (13)

G, differentiation grade; G1-3, low, moderate or intermediate, high grade. ER, estrogen receptor alpha; PgR, progesterone receptor; T, tumor size; pT1, 0–2 cm; pT2, 2–5 cm; pT3, > 5 cm; pT4, ulcerated or attached; Tis, carcinoma in situ; pTX, primary tumor of unknown pT; N, node status; LVI, lymphovascular invasion; n, number of cases.

### Pathological features

Diameter of the tumors was measured in millimeters, their grading (G) was classified as standard G1, G2, G3; node involvement was defined as positive (N ≥ 1) (cancer cells found in one or more lymph nodes) or negative (N = 0) (absence of regional metastases). We further evaluated lymphovascular invasion (LVI), classified as positive or negative according to the presence or absence of tumor cells in the lumens of lymphatic or blood vessels (Table. 1).

### Biological features

In all cases, serial-step 5 μm sections were cut from paraffin-embedded tissue samples and stained with hematoxylin-eosin for histological examination. ERα and progesterone receptor (PgR) status was determined by immunohistochemistry (IHC). Tumors expressing at least 1% of cells positive for ERα or PgR were considered positive, according to recommendations of 10^th ^St Gallen Conference on Primary Therapy of Early Breast Cancer [31]. IHC staining for the replicative cell fraction was performed using a Ki-67 monoclonal antibody (mAb) (DAKO, Denmark). Ki-67 expression results were arbitrarily classified as low (≤ 15% of stained cells), medium (16–25%) or high (> 26%). HER2 levels were determined by IHC using the HercepTest (DAKO). HER2 expression levels obtained by IHC were scored as 0 (no staining), 1+ (faint incomplete membranous pattern), 2+ (moderate complete membranous pattern) and 3+ (strong membranous pattern). Samples with scores 0 and 1+ were considered HER2-negative and with the score 3+ were considered HER2-positive. To confirm or exclude HER2 positivity, samples with a score 2+ were further evaluated with fluorescence in situ hybridization (FISH) using PathVysion assay (Abbott Diagnostics, Rome, Italy). The status of p53 has not been assessed. The characteristics of patients and tumors are summarized in Table. 1.

### Detection of leptin and ObR in breast cancer biopsies

The expression of leptin and ObR was investigated by IHC with specific Abs, as described by us before [18]. Briefly, tissue sections were deparaffinized using a dry oven at 60°C overnight, then the slides were dewaxed in xylene and rehydrated in graded series of ethanol. After rinsing in PBS, endogenous peroxidase activity was inhibited by incubation with 30% hydrogen peroxide, diluted in 100% methanol for 30 min at 4°C. After three washes in PBS the sections were incubated with 1.5% blocking serum for 1 h, then the sections were incubated for 3 h with primary antibodies. For leptin staining, we used the A20 leptin polyclonal Ab (pAb) (Santa Cruz Biotechnology, Santa Cruz, USA) at 1:100 dilution; for ObR staining, the M18 ObR pAb (Santa Cruz Biotechnology) at 1:50 dilution overnight at 4°C. The leptin and ObR antigens were detected with avidin-biotin-peroxidase ABC staining systems (Santa Cruz Biotechnology). All slides were counterstained with Mayer's hematoxylin. Breast specimens previously classified as positive for the expression of the studied markers were used for control and protocol standardization. In negative controls, primary Abs were omitted. Assessment of immunoreactivity was performed in at least 10 different section fields by two independent evaluators by light microscopy, and the mean percentage of tumor cells displaying positive staining was scored. The expression of leptin and ObR in cancer samples was classified using a four-point scale: 0, < 10% stained cells; 1+, 10–50% cells with weak staining; 2+, >50% cells with weak staining; 3+, > 50% cells with strong staining. Tumors with the score of at least 1+ were considered positive for the expression of leptin or ObR.

### Cell culture and treatments

Breast cancer cell lines used in this study included MCF-7, SK-BR-3, BT-474 and ZR-75-1 cells, all purchased from the American Type Culture Collection (Rockville, MD, USA). MCF-7 cells were grown in Dulbecco's modified Eagle's medium (DMEM:F12) (Cellgro, Herndon, VA, USA) containing 5% calf serum (CS) and 1% Penicillin/Streptomycin (P/S) (Cellgro). SK-BR-3 cells were grown in DMEM F12 containing 10% fetal bovine serum (FBS) and 1% P/S. BT-474 cells were grown in DMEM:F12 containing 10% FBS, 1% P/S and 0.01 μg/mL insulin. ZR-75-1 cells were grown in RPMI-1640 (BioWhittaker, Walkersville, MD, USA) containing 10% FBS, 1% P/S and 1 mM sodium pyruvate (Cellgro).

For growth factor stimulation, the cells (4.5 to 6.0 × 10^5 ^cells/100 mm dish) were placed in phenol red-free serum-free medium (SFM) [32] for 24 h and then stimulated with different doses of leptin (Ob) (R&D systems, Minneapolis, MN, USA) or epidermal growth factor (EGF) (BD Biosciences, Bedford, MA, USA) for 15 min.

### Western blotting (WB) and immunoprecipitation (IP)

Total and phospho-HER2 levels were detected by Western Blotting (WB) with Neu C-18 and p-Neu Tyr1248-R Abs (Santa Cruz Biotechnology), respectively. Total protein lysates were obtained as described before [32]. For WB or IP of ObR, we used H-300 Ab (Santa Cruz Biotechnology) recognizing a common domain within ObRl and ObRs and suitable for detection of all ObR isoforms. For WB, we routinely used 35–100 μg of total protein, while for IP, 500 μg of proteins were precipitated using protein A agarose beads (Sigma-Aldrich, St. Louis, MO, USA). IP samples were precleared with protein A agarose for 4 h before addition of primary Abs. Unrelated IgG was used as negative control for ObR Abs. Otherwise, all WB and IP procedures and measurements of protein abundance followed protocols described in detail before [32]. All WB and IP/WB experiments were repeated at least 3 times.

### HER2 and ObR detection by immunofluorescence/deconvoluted microscopy

MCF-7 cells were plated in 2-well Permanox chamber slide (Nunc, Rockester, NY, USA) at a concentration 9 × 10^5 ^cells/well. Next day, the cultures were shifted in SFM for 24 h and then treated with leptin 100 ng/mL for 15 min. Then the cells were washed three times with PBS, fixed in 4% paraformaldehyde for 20 min at 4°C, washed again and blocked with 7.5% BSA for 2 h at room temperature. The expression of HER2 was detected using HER2 Neu C-18 Ab 1:100 (Santa Cruz) and donkey anti-rabbit IgG-TRITC 1: 1000 (Santa Cruz); ObR was detected using Ob-R F-18 Ab 1:50 (Santa Cruz) and donkey anti-goat IgG FITC 1:1000 (Santa Cruz). The slides were covered with Vectashield mounting medium containing DAPI (Vector laboratories, IncBurligame, CA, USA) to allow visualization of cells nuclei. The coexpression of HER2 and ObR was assessed using Olympus IX81 deconvoluted microscope and Slidebook software.

### Elisa for phospho-HER2

MCF-7 cells were stimulated with 50, 100, 200, 500 ng/mL leptin for 15 min, or were left untreated in SFM. Total proteins were isolated as described for WB. Tyrosine phosphorylated HER2 (Tyr 1248) was measured using DuoSet IC Human Phospho-ErbB2 Elisa kit (R&D Systems, Minneapolis, MN, USA), following manufacturers instructions. 350 μg of sample proteins and 150 ng of the provided Tyr 1248 HER2 protein control were used for measurements. The reading was done using Microplate Autoreader Bio-Tek EL311.

### Statistical analysis of data

Relationships among markers studied by IHC were evaluated using Fisher's exact test for count data with a significance level of 0.05 [33]. Statistical analyses were performed using R for Windows software (R Development Core Team. R: A language and environment for statistical computing. R Foundation for Statistical Computing, Vienna, Austria, 2007. ISBN 3-900051-07-0, URL ). All Elisa and WB assays were done data in triplicate and the results were evaluated using one-way analysis of variance.

## Results

### ObR and HER2 are coexpressed in breast cancer cell lines

**Figure 1:**
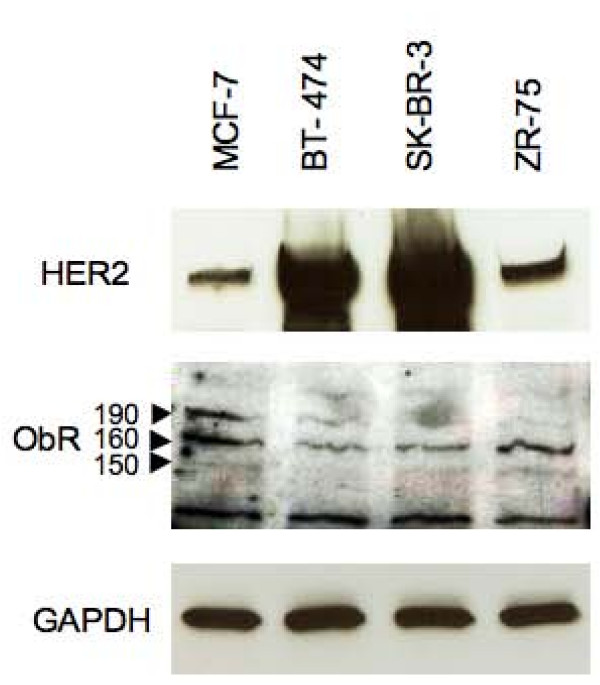
**Expression of HER2 and ObR in breast cancer cell lines**. The expression of HER2 (185 kDa) and different isoforms of ObR (150–190 kDa, indicated by arrows) was detected in 50 μg of total protein lysates obtained from MCF-7, BT-474, SK-BR-3, and ZR-75 cell lines by WB with specific Abs, as described in Materials and Methods. The presence of a constitutively expressed enzyme GAPDH was assessed in the same blot as control of protein loading.

**Figure 2:**
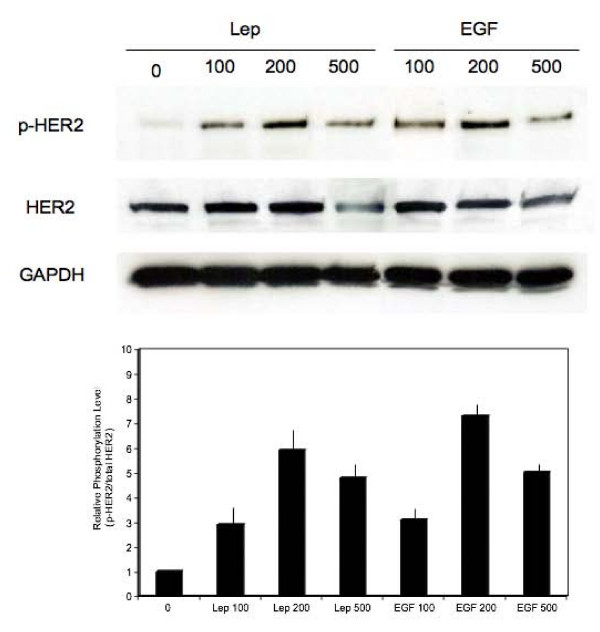
**Transactivation of HER2 by leptin**. MCF-7 cells were stimulated for 15 min with 100, 200, 500 ng/mL doses of leptin (Lep) or EGF. The expression of Tyr 1248 HER2 (p-HER2), total HER2 levels (HER2) was studied in 100 μg of total proteins by WB with specific Abs, as described in Materials and Methods. The levels of GAPDH in the same blots were assessed to control protein loading. The graph represents levels of Tyr 1248 HER2 relative to total HER2 under different stimuli. Bars represent SE.

The results obtained in HEK 293T kidney cells engineered to overexpress ObR and HER2 suggested that leptin can transactivate HER2 [22]. Thus, we examined whether similar interactions could occur in breast cancer cell models. To this end, we tested ObRl and ObRs expression in four different cell lines with varying levels of HER2: BT-474 and SK-BR-3 cells, known to express high levels of HER2, and MCF-7 and ZR-75 cell lines characterized by moderate HER2 expression (Figure. 1). The expression of the signaling ObRl isoform (~190 kDa) as well as two short ObR isoforms (~150 and 160 kDa) was confirmed in MCF-7 cells. Low levels of ObRl were also found in BT-474 cells, while minimal expression of ObRl was detected in SK-BR-3 and ZR-75 cells. All cell lines expressed different isoforms of ObRs (Figure. 2).

### Leptin treatment transactivates HER2

To study whether leptin can transactivate HER2, we focused on MCF-7 cell line as it expresses both HER2 and high levels of ObRl and ObRs ([32] and Figure. 1). As demonstrated by us and others before, MCF-7 cells respond to 100–500 ng/mL leptin with the activation of different intracellular pathways leading to cell proliferation and survival [32,34].

The acute stimulation (15 min) of MCF-7 cells with leptin induced HER2 phosphorylation on Tyr1248. Leptin-dependent activation of Tyr1248-HER2 was the strongest with 200 ng/mL leptin, but HER2 was found phosphorylated also with 100 and 500 ng/mL leptin (Figure. 2). The highest doses of leptin (500 ng/mL) induced rapid downregulation of HER2, most likely due to ligand-dependent internalization [35]. Similar induction of Tyr1248-HER2 was observed with EGF, a known activator of this receptor [36] (Figure. 2). Like with leptin, the best stimulation of HER2 was seen with the 200 ng/mL dose and high EGF concentrations produced HER2 downregulation. Lower doses of leptin or EGF (10, 25 and 50 ng/mL) did not induce any HER2 response in MCF-7 cell model (data not shown).

Activation of HER2 by leptin was also assessed independently using a specific phospho-HER2 Elisa kit. With this methodology, we found that leptin at 100, 200, and 500 ng/mL induced HER2 phosphorylation on Tyr1248 by 135, 230, and 85%, respectively.

### ObR and HER2 colocalize and coprecipitate in breast cancer cells

**Figure 3:**
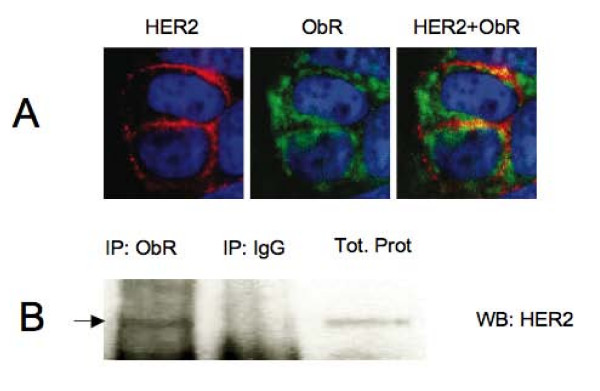
**Physical interactions between ObR and HER2**. (A) Growing subconfluent cultures of MCF-7 cells were processed for HER2 (red staining) and ObR (green staining) immuofuorescence as described in Materials and Methods. Colocalization of HER2 and ObR was detected by merging (HER2+ObR) images (yellow staining). Cell nuclei were detected by DAPI (blue staining). (B) Total proteins from growing MCF-7 cells were immunoprecipitated with ObR Abs or control unrelated IgG, as described in Materials and Methods. The presence of HER2 in ObR and IgG IPs was detected by WB and is indicated by arrow. 35 μg of total MCF-7 cell proteins were run on the same gel to control HER2 Ab specificity.

Next, we probed if HER2 and ObR can physically interact in breast cancer cells. Using specific immunofluorescence staining combined with confocal microscopy, we found that HER2 colocalizes with ObR in MCF-7 cells (Figure. 3A). The colocalization was detected in 20 ± 0.7% of cells. In addition, we found that HER2 can be detected in ObR immunoprecipitates obtained from growing MCF-7 cells (Figure. 3B).

### The leptin/ObR system is coexpressed with HER2 in a large subgroup of breast cancers

**Figure 4:**
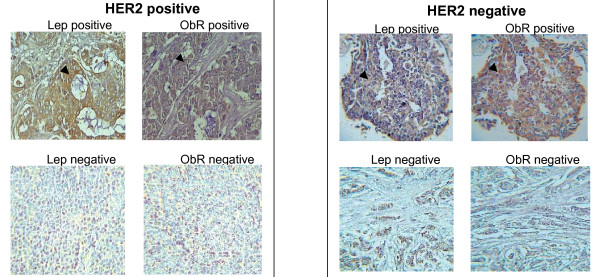
**Expression of the leptin/ObR system in HER2-positive and -negative breast cancer**. The expression of leptin and ObR was studied by IHC, as described in Materials and Methods in HER2-positive and HER-2-negative samples. The magnification level is 40x. Arrows indicate the example areas of leptin and ObR staining.

The data obtained in breast cancer cell models prompted us to investigate whether ObR isoforms together with their ligand, leptin, can be coexpressed with HER2 in human breast cancer. We analyzed the expression of leptin and ObR by IHC in HER2-positive and HER2-negative breast cancers characterized in Table. 1. This screening demonstrated that both leptin and ObR can be expressed in both HER2-positive and HER-2-negative tumors (Table. 2, Figure. 4).

**Table 2:** Associations between leptin and ObR in HER2-positive and HER2-negative breast cancer

		***ObR +***		***ObR -***		***p-value***
		***N***	***%***	***N***	***%***	

***All tumors***	Leptin +	46	78.0	1	1.7	< 0.001
	Leptin -	3	5.1	9	15.3	
						
		N	%	N	%	
***HER2 ***	Leptin +	23	74.2	0	0.0	< 0.001
***positive***	Leptin -	1	3.2	7	22.6	
***HER2 ***	Leptin +	23	82.1	1	3.6	0.045
***negative***	Leptin -	2	7.1	2	7.1	

The expression of leptin and ObR was assessed by IHC, as described in Materials and Methods. The percentage (%) and actual number (N) of tumors expressing combinations of leptin and ObR is given for all tumors as well as in HER2-positive and -negative subgroups. Associations between leptin and ObR in were evaluated using Fisher's exact test for count data.

As noted in previous studies from our and other laboratories, leptin and ObR tend to be coexpressed in different cancers [18,19,37,38]. Similarly, the present analysis confirmed strong and significant associations between leptin and ObR in all tumors (Table. 2 and Figure. 4); there were only 4 cancers with a discordant result so that the overall agreement was higher than 93%. The coexpression of leptin and ObR was confirmed both in HER2-positive and in HER2-negative subgroups; the overall agreement was 97% and 89% respectively (Table. 2).

The expression of leptin or ObR was, at least numerically, associated with tumor size, being more frequent in large (> 10 mm) tumors (Table. 3); however, owing to the limited sample size, this relationship was only marginally significant for leptin (*p *= 0.061) and even less significant for ObR (*p *= 0.137). The expression of leptin or ObR was not associated with other considered variables (Table. 3).

**Table 3:** Associations between leptin, ObR, leptin/ObR and different clinicopathological parameters

			**Leptin**		**ObR**	
			
		**N**	**Positive (%)**	**p-value**	**Positive (%)**	**p-value**
***Diameter (mm)***				0.061		0.137
	≤ 10 mm	9	55.6		66.7	
	> 10 mm	42	85.7		88.1	
***N***				> 0.5		0.242
	N0	19	84.2		94.7	
	N+ (N > 0)	33	78.8		81.8	
***LVI***				> 0.5		0.473
	Negative	24	79.2		87.5	
	Positive	27	77.8		77.8	
***Menopausal status***				> 0.5		> 0.5
	Postmenopausal	15	80.0		86.7	
	Premenopausal	39	82.1		84.6	
***Histotype***				> 0.5		0.432
	Other	3	66.7		66.7	
	Ductal (invasive)	45	80.0		84.4	
	Inflammatory	4	100.0		100.0	
	Intraductal	3	66.7		66.7	
	Lobular (invasive)	4	75.0		75.0	
***G***				0.495		0.366
	G1	7	85.7		100.0	
	G2	18	88.9		88.9	
	G3	26	73.1		76.9	
***Ki-67***				> 0.5		> 0.5
	0–15%	33	75.8		81.8	
	16–25%	9	88.9		88.9	
	26–100%	14	85.7		85.7	
***ER/PgR***				> 0.5		> 0.5
	ER-/PgR-	12	75.0		83.3	
	ER-/PgR+	1	100.0		100.0	
	ER+/PgR-	6	83.3		83.3	
	ER+/PgR+	37	81.1		83.8	
***T***				0.019		0.400
	pT1	20	70.0		80.0	
	pT2	23	91.3		91.3	
	pT3	2	0.0		50.0	
	pT4	8	100.0		87.5	
	pTx	1	100.0		100.0	
	TIS	3	66.7		66.7	
***HER2***				0.342		0.306
	Negative	28	85.7		89.3	
	Positive	31	74.2		77.4	

For each level of each clinicopathological variable, the total number (N) of patients is given together with the percentage of subjects positive for leptin or ObR expression. Abbreviations as in Tab. 2.

## Discussion

Obesity increases the risk of postmenopausal breast cancer by 30–50% [2]. Furthermore, excess body weight is associated with poorer survival and increased recurrence of cancer, regardless of menopausal status, after adjustment for stage and treatment [2]. Because of the increasing number of obese breast cancer patients, the mechanism of this phenomenon is currently under thorough investigation. Multiple studies implicated different biologically active substances produced by adipose tissue as possible contributing factors [4,5,7]. Steroid hormones, e.g., estrogens, or growth factors, e.g., insulin-like growth factor I, all of which are secreted by fat cells, are known to promote breast cancer cell growth, transformation and survival [39,40]. New evidence obtained in cellular and animal breast cancer models suggests that leptin, the major hormone produced by the fat tissue, can be mechanistically involved in these neoplastic processes [6].

Although, in view of some inconsistent reports [5,6,21,41,42], the impact of circulating leptin needs further evaluation, one recent study correlated coexistence of high systemic leptin concentrations and high intratumoral ObR mRNA levels with poor prognosis in breast cancer patients [21]. In addition, breast cancer cells themselves can synthesize leptin in response to obesity-related stimuli [18,43,44]. It remains to be evaluated if the frequent coexpression of leptin and ObR in breast tumors [18-21,45] indeed reflects patient's adiposity.

As shown by many authors, leptin can exert its action not only through ObR, but also through many other signaling systems [5,6]. In the context of the most aggressive breast cancer, it is important that leptin could crosstalk with HER2 either through ObR, HER1, and JAK2 [22,29].

HER2 is a major marker of aggressive breast cancer and an important pharmaceutical target [28,46]. HER2-targeted therapies with trastuzumab improved survival of patients with HER2 overexpressing metastatic breast cancer and early-stage breast cancer. However, primary or treatment-induced resistance to this drug often occurs [28,47]. The mechanisms of this resistance seem to include increased activation of other signaling systems, for instance, overexpression of IGF-I receptors, increased synthesis of EGF or TGF-alpha, mutation of PTEN phosphatase resulting in constitutive PI-3K activation [28,47]. Thus, targeting alternative signaling systems in HER2-positive tumors might prove beneficial; indeed, clinical trials exploring such options are underway [28,48].

However, the interaction between HER2 and the leptin system has not been well explored in breast cancer. Here we report that in breast cancer cell lines, endogenous coexpression of HER2 and ObR is common, but cells expressing very high levels of HER2 appear to express low levels of ObR. In MCF-7 cells, which contain relatively high levels of ObR [32] and moderate levels of HER2, high physiological doses of leptin can induce HER2 tyrosine phosphorylation. Similar transactivation was recently described in SK-BR-3 cells [29]. In the case of MCF-7 cells, we show that leptin-dependent stimulation of HER2 could be facilitated by proximity or direct interaction of HER2 and ObR, as demonstrated by colocalization and coimmunoprecipitation experiments. The exact mechanism of HER2 phosphorylation by liganded ObR is not known, but one could speculate that intermediate cellular tyrosine kinases, such as JAK2 (normally binding to activated ObRl) or src (possibly interacting with either ObRl or ObRs), could be involved. On the other hand, in cells expressing low ObRl levels, e.g., SK-BR-3 cells, leptin appears to transactivate HER2 via HER1 and JAK2 pathways [29].

In this study, we evaluated whether HER2/ObR crosstalk observed in cellular models could occur in human breast cancer in vivo. We noted that the leptin/ObR system is coexpressed with HER2 in a large fraction of breast cancers, which supports the possibility of intratumoral ObR/HER2 interactions. Notably, the presence of leptin/ObR was numerically more frequent in larger tumors. However, perhaps due to the relative small sample of tumors analyzed, we were unable to detect any associations between leptin/ObR and either tumor grade, ERα/PgR, or metastasis, reported by different authors previously [18-20].

The statistical analysis employed deserves some comments. The Fisher's exact test is the dominant method for making inferences from 2 × 2 tables where the number of observations is small. The test assumes that both of the margins in a 2 × 2 table are fixed by construction ("conditional" test), but if an alternative process generates the data, the test might not provide a correct coverage. Nonetheless, the Fisher's exact test is often used, since in general, it generates a conservative result. In our case no margins of the 2 × 2 tables were fixed, so that an "unconditional" exact test would be more appropriate. To validate our analysis, we performed such test, which gave, as expected, a more significant result, in particular when analyzing the association between leptin and ObR in HER2-negative tumors (*p *= 0.024 vs. *p *= 0.045 obtained using the Fisher's exact test).

The finding that both leptin and ObR can be found not only in HER2-positive but also in HER2-negative tumors suggests that leptin/ObR and HER2 systems are controlled by separate mechanisms. Interestingly, in our latest screening of ~90 "triple-negative" tumors (lacking ERα, PgR, and HER2 expression), we detected leptin and ObR in most cases analyzed (manuscript in preparation, data not shown). In this cellular context where hormonal and traditional targeted therapy are not applicable, the leptin system might constitute a new attractive target.

## Conclusion

In summary, our results suggest the existence of crosstalk between HER2 and the leptin system in breast cancer. This notion implicates targeted anti-ObR therapies as possible future therapeutic options, especially in tumors that become resistant to targeted HER2 therapy. Such therapeutic approaches could be especially effective in patients expressing high physiological levels of leptin (100–300 ng/mL), characteristic for the overweight and obese phenotype.

## Competing interests

The authors declare that they have no competing interests.

## Authors' contributions

EF and AM carried out molecular and IHC studies, contributed to manuscript writing and editing; MT, AA, VP, and B DS carried out IF and leptin/ObR IHC studies; AR and MF B prepared pathology samples and evaluated IHC for different markers; RM performed statistical analysis of all data; AM M, GL C and AG participated in the design of the study and edited the manuscript; ES conceived and coordinated the study and drafted the manuscript.

## Pre-publication history

The pre-publication history for this paper can be accessed here:


